# Selecting, implementing and evaluating control and placebo conditions in light therapy and light-based interventions

**DOI:** 10.1080/07853890.2023.2298875

**Published:** 2024-02-08

**Authors:** Manuel Spitschan

**Affiliations:** a Max Planck Institute for Biological Cybernetics, Translational Sensory & Circadian Neuroscience, Tübingen, Germany; b Technical University of Munich, TUM School of Medicine and Health, Chronobiology & Health, Munich, Germany; c Technical University of Munich, TUM Institute for Advanced Study (TUM-IAS), Garching, Germany

**Keywords:** Light therapy, light-based interventions, ipRGCs, α-opic, light, placebo, control conditions, bright light therapy

## Abstract

**Introduction:** Light profoundly influences human physiology, behaviour and cognition by affecting various functions through light-sensitive cells in the retina. Light therapy has proven effective in treating seasonal depression and other disorders. However, designing appropriate control conditions for light-based interventions remains a challenge.

**Materials and methods:** This article presents a novel framework for selecting, implementing and evaluating control conditions in light studies, offering theoretical foundations and practical guidance. It reviews the fundamentals of photoreception and discusses control strategies such as dim light, darkness, different wavelengths, spectral composition and metameric conditions. Special cases like dynamic lighting, simulated dawn and dusk, complex interventions and studies involving blind or visually impaired patients are also considered.

**Results: **The practical guide outlines steps for selection, implementation, evaluation and reporting, emphasizing the importance of α-opic calculations and physiological validation.

**Conclusion:** In conclusion, constructing effective control conditions is crucial for demonstrating the efficacy of light interventions in various research scenarios.

## Introduction

Light has a profound influence on human physiology, behaviour and cognition. Through a pathway connecting the fine layers of photosensitive cells in the eye, the retina, to various retinofugal targets, light affects various physiological functions, including the circadian clock and melatonin suppression. The use of light therapy, also called bright light therapy (BLT), has been established as an effective method to mitigate and treat seasonal affective disorder (SAD), non-seasonal major depressive disorder [1], bipolar depression [2], and other psychiatric and neurological disorders [3–7].

Research on the efficacy of light-based interventions typically compares an active light condition, e.g. bright light at a corneal illuminance of 10,000 lx, against a control or placebo condition designed or purported to be ineffective in stimulating the biological pathway assumed to be underlying the positive treatment effects. Understanding and evaluating the impact of the intervention requires adequate control conditions. In the literature, different strategies have been used for these control or placebo conditions, including variations in intensity (‘dim light’), wavelength (‘red light’), spectral tuning (‘amber light’) or a combination of these (‘dim red light’). To date, there is no guidance on appropriate control/placebo conditions. Here, we present a systematic framework for selecting, implementing and evaluating suitable control/placebo conditions in studies using light or light exposure as the primary intervention.

This article aims to provide a theoretical grounding for control conditions and offer practical guidance. Where applicable, additional references are given for novices in the field. We will first review the fundamentals of photoreception, providing insights into mechanisms underlying light interventions. We will then review common strategies for control conditions. Finally, we will provide a practical guide for selecting, implementing, evaluating and reporting light interventions.

## Fundamentals

### How do photoreceptors sense light? Photoreception and the principle of univariance

Photobiological effects arising from ocular light exposure are due to photons being captured by photoreceptors in the retina and the fine layer of nerve cells in the back of the retina. The retina contains cones and rods, considered the ‘canonical’ photoreceptors underlying vision and visual perception, including colour, motion and spatial vision. There are three classes of cones that are different in their wavelength preference, technically called spectral sensitivity: The long-wavelength-sensitive L cones, the medium-wavelength-sensitive M cones, and the short-wavelength-sensitive S cones. The rods differ from the cones in shape (as given by their name) and in terms of the operating range, as they only signal light in dark to dim conditions and saturate and are non-functional under most daylight conditions. In addition to the cones and rods, a proportion of downstream neurons in the retina, the retinal ganglion cells (RGCs), are also *directly* light sensitive by expressing a photosensitive protein, melanopsin, in their cell body and processes [8–14]. Only <5% of RGCs are intrinsically photosensitive in this way (abbreviated as ipRGCs).

All photoreceptors – cones, rods, ipRGCs – are sensitive due to the expression of photosensitive pigments that capture photons and convert them into neural impulses. The photoreceptors’ spectral sensitivity is determined by the pigment’s biochemical properties, with cones, rods and ipRGCs having distinct but overlapping spectral sensitivities. Importantly, when the pigment absorbs photons, the pigment loses information about the wavelength of the light, as only a univariant signal – the photoreceptor output – is produced. This is the *principle of univariance* [15]: a single photoreceptor (class) cannot distinguish between differences in wavelength and differences in intensity, as it only produces a univariant output (from a multidimensional input of photons at different wavelengths). The principle of univariance is key for selecting and designing control conditions, as different spectra of light can produce the same effect at the level of the photoreceptor(s).

The signals generated by the different photoreceptor classes leave the retina through a range of different pathways. For the non-visual, circadian and neuroendocrine effects of light, this includes the retinohypothalamic pathway, connecting the retina to the suprachiasmatic nucleus (SCN) in the hypothalamus. More recently, there has also been evidence from animal models for a direct pathway connecting the ipRGCs to neural circuits controlling mood, representing a candidate neural substrate for light-therapeutic effects.

#### Further reading

Do [8] and Spitschan [14].

### How do we quantify the effect of light on the photoreceptors? From retinal mechanisms to metrology

Light is the visible part of the electromagnetic spectrum, between 380 nm and 780 nm. We can describe light using terms of physics. The most complete representation of light in physical terms is the spectrum or spectral power distribution, which gives the amount of energy – or, equivalently, the number of photons – at a specific wavelength. Spectral power distributions of light are measured using spectroradiometers. To make this physical description of light relevant in light interventions, we need to convert it to a physiologically relevant quantity.

Each photoreceptor class has a distinct spectral sensitivity. Converting a physically complete representation of light – the spectral power distribution – into the signals available or, more accurately, produced by the photoreceptor class requires knowledge of these spectral sensitivities. Over the past few decades, careful biological and physical measurements have established the spectral sensitivities of the cones, rods and the melanopsin component of the ipRGCs. This knowledge has recently been translated to an International Standard, CIE S 026/E:2018 [16], which describes standard spectral sensitivity functions for converting spectra into photoreceptor activations. These spectral sensitivity functions, called α-opic spectral sensitivities, where α corresponds to L-cone-opic, M-cone-opic, S-cone-opic, rhodopic and melanopic, are implemented in a range of tools that allow for the calculation of the α-opic irradiances, including the CIE Toolbox and the open-access, open-source web platform *luox* [17] available at https://luox.app/. The metrology and quantifications developed within CIE S026 offer a systematic method for evaluating the appropriateness of control/placebo conditions in light-based interventions: Not only does it allow us to express light in physiologically relevant terms, i.e. concerning the photoreceptors, we aim to stimulate in light interventions, but we can also use its quantities to describe differential activation between active vs. control conditions. This is further developed in the *Practical guide*, under *Step 3: evaluation, α-opic calculations*.

#### Further reading

Price and Blattner [18], Schlangen and Price [19] and Spitschan et al. [20].

### What are criteria for control conditions?

To examine the effects of light on any function requires the adequate design of a control condition. Control conditions form a key part of good experimental design. In clinical trials, placebo conditions, which look and feel the same to a participant as the active condition under investigation, are an essential part of establishing the efficacy of, e.g. an active compound, agent or ingredient. In pharmacological studies using pills or tablets, this can be done by simply administering pills without the active ingredient. For more complex interventions, including behavioural ones, designing control conditions becomes challenging.

For light interventions, we face a particular challenge: a participant will see and perceive the light exposure and possible differences between ‘active’ and ‘control’ conditions under most circumstances. This is not a new problem, and has been discussed extensively in the light-therapy literature [21–27] (also see [28] for an autobiographical accounton the development of control conditions in light therapy). Metameric lights (discussed below under Section ‘*Metameric pairs and silent-substitution modulations*’), which are matched in visual appearance but stimulate melanopsin differently, provide a novel and innovative way of eliciting the non-visual effects of light.

In general, we can distinguish between absolute and relative control conditions. Absolute control conditions are useful for establishing whether light affects a specific function at all. This would include, e.g. bright light exposure compared against a dim-light or dark control. Relative control conditions are more specific, creating a differential stimulation scenario, e.g. does wavelength A affect a specific function more than wavelength B. We return to these differences when we discuss selecting control conditions in the *Practical guide* under Section ‘*Step 1: Selection*’.

#### Further reading

For further reading, Terman [26] and Eastman [24].

## Strategies for control conditions

### Dim-light controls

The most common control condition in studies using light is the dim-light control. Typically, the dim-light control condition employs light conditions that are below some criterion light level corresponding to an assumed, or purported, ineffective intensity. The dim-light control is also used in the dim-light melatonin onset, a biomarker indicating the onset of melatonin secretion under dim-light conditions. In the literature, the term ‘dim light’ is amorphous. A meta-analysis investigating the meaning of ‘dim light’ has found values ranging across several orders of magnitude [29], indicating a large variability in how the term ‘dim light’ is understood.

### Darkness controls

A more substantial control going in the same direction as the dim-light control is to use total darkness. Such a condition will provide an absolute control, in the sense that it truly establishes a zero-condition. Unfortunately, it is doubtful that such a strategy is feasible, tolerable or acceptable to participants for long periods of time. In addition, such a condition imposes substantial logistical demands on how an experimenter interacts with the participant in total darkness.

### Different-wavelength controls

When the ‘active’ condition comprises light of a single wavelength, a common strategy is to select a different wavelength for control condition. With different-wavelength controls, it is key to understand by which criterion the light of different wavelengths is matched. For wavelength stimuli targeted to preferentially activating the melanopsin system, this could be, for example, matching irradiance/radiance, i.e. the total energy, or matching illuminance/luminance, i.e. the intensity of light as seen by the photopic luminosity function (equivalent to a weight sum of the the L and M cones).

### Spectral composition controls

In studies examining the effect of spectra with an enhanced short-wavelength content, a common strategy is to compared this against a spectrum matched in some criterion, e.g. luminance/illuminance, but with a different spectrum or a proxy of spectrum, such as correlated colour temperature (CCT) and chromaticity. Using common spectral tuning strategies, lights differing in CCT will, in most cases, produce a difference in melanopsin activation but also stimulate the cones differently – a fact trivially demonstrated by the fact that the colour appearance is different.

### Metameric pairs and silent-substitution modulations

Metameric lights are pairs of lights, which are matched in the amount that they excite the cones, thereby nominally appearing equivalent to an observer. Because the spectral sensitivity of melanopsin differs from the cones, metameric pairs generally stimulate melanopsin differently, thereby creating a condition in which melanopsin is selectively stimulated, with no differential stimulation of the cones. Metameric pairs have been used to understand the impact of light on NIF function [30–34], demonstrating the importance of melanopsin in these functions. Metameric lights can be generated using the method of silent substitution [35,36], and convenient computational techniques exist to generate these modulations.

The method of silent substitution is a general technique for generating pairs of lights that stimulate a class of photoreceptors selectively without stimulating another class of photoreceptors. In principle, all photoreceptor classes can be stimulated selectively using this technique. In silent-substitution modulations, there is an active condition and a background. The difference in activation between background and modulation is typically expressed as a ratio or as contrast (see Section ‘α-opic calculations’).

### Modulating light exposure timing

The effect of light exposure depends on time of day. Light in the evening suppresses the production of melatonin [37–40] and delays the circadian clock, while light in the morning advances the circadian clock [41–43]. This is encapsulated in the phase response curve (PRC) for light. Some light interventions target specifically the timing of the circadian clock, including advanced sleep phase disorder (ASPD) and delayed sleep phase disorder (DSPD) [44,45]. If the goal of an intervention is to shift the circadian clock one way or another, then a comparator condition could change light timing. For example, exposure to bright morning light might advance circadian rhythms in individuals with delayed melatonin rhythms, and light exposure at another time could serve as a control. As PRCs are typically measured under specific conditions, it is nontrivial to predict the size of a shift of the circadian clock at a given circadian time for arbitrary light conditions.

### Non-photic controls

In this review, we focus on light exposure as an intervention but circadian physiology is subject to non-photic influences, including meal timing, exercise and social contact [46–49]. These could serve as non-pharmacological comparator conditions. Negative air ionizers have been used as controls in clinical trials of BLT [50–54], with demonstrated effects on mood [55], though the underlying mechanisms of action are considerably less clear than for BLT.

## Special cases

So far, we have considered relatively standard cases of light interventions. In the following, we will consider additional cases, which pose a specific challenge for developing control conditions.

### Dynamic lighting interventions

Previously, we have not considered light interventions, which are extended and changing over time. The use of dynamic light over multiple hours or days is an emerging area of research, mirroring to some degree a commercial trend to deliver ‘biodynamic’ or ‘human-centric’ light solutions (in fact some of the cited papers are industry-sponsored). These dynamic lighting interventions typically implement changes in spectral composition or CCT, intensity, or both. It is not immediately evident what an appropriate control condition may be to examine specifically the dynamic component of the intervention. As the goal of study is to examine the effect of an active condition *relative* to a control condition, one can imagine several different strategies for specifying the control condition: match in total photon catch, match average intensity, match in average CCT, or similar.

### Simulated dawn and dusk interventions

Related to dynamic lighting interventions, but predating them by a few decades is the use of simulated dawn or dusk transitions [50,56–74] around wake-up and sleep time. The logic of dawn/dusk simulations is to make light exposure at these key times similar to the natural progression of dawn/dusk given by the availability of daylight and twilight.

### Complex interventions

It is conceivable to wish to test a complex intervention, which differs not just in one stimulus dimensions (see Section ‘Step 1: selection’ and Section ‘Step 2: implementation’ for a discussion). A pragmatic example is the use of daylight received through windows vs. an electric lighting system enhancing spectral power distribution. In these cases, a careful formal analysis must be conducted to characterize to what extent and along which dimensions these conditions differ.

### Studies with blind or visually impaired patients

We stated earlier (see Section ‘What are criteria for control conditions?’) that a key challenge to designing appropriate control/placebo conditions is the fact that light is a visual stimulus and so it is near-impossible to create stimulus conditions to which participants are blind, with the limited exception of the use of metameric stimuli (see *Metameric lights*). In some blind individuals, the non-visual effects of light are preserved, indicating an intact melanopsin system in the absence of cones and rods [75–82]. In some conditions, such as congenital achromatopsia, there is cone-specific dysfunction [83]. Very few studies have been on light therapy in blind individuals [84].

## Practical guide

### Step 1: selection

The selection of control conditions depends on the exact research question, as well as the target active condition. We list different classes of research questions, appropriate target active condition and possible controls in Table 1. In some cases, it is advisable to deploy both an *absolute* and a *relative* control. The first establishes that the two conditions under investigations are biologically effective at all, and the second allows for the measurement between any differences elicited by these conditions. In practice, this means including a dim-light condition in all experiments.

**Table 1.** Criteria for selection of conditions, organized by research question.

Research question	Possible active condition	Adequate control condition	Type of control	Note
Is X affected by light?	Bright light with no specific spectral requirements	Dim light Darkness	Absolute	Measurement of parametric dose–response to intermediate light conditions is advised
Is wavelength A more effective in producing X than wavelength B?	Wavelength A	Wavelength B	Relative	Need to establish some criterion by which wavelength A and wavelength B are matched in exposure (e.g. irradiance/radiance, illuminance/luminance)
Is spectrum A more effective in producing X than spectrum B?	Spectrum A	Spectrum B	Relative	Need to establish some criterion by which spectrum A and spectrum B are matched in exposure (e.g. irradiance/radiance, illuminance/luminance)
Does photoreceptor class A contribute to/drive X?	Modulation spectrum targeted to maximize contrast on photoreceptor class A	Background/reference spectrum	Relative	Limited contrast

### Step 2: implementation

Assuming that the control/placebo condition differs only in one dimension – e.g. spectral power distribution, wavelength, intensity, spatial distribution, temporal pattern – special care must be taken to truly introduce no inadvertent changes in other light parameters.

### Step 3: evaluation

#### Spectroradiometric measurements

**Figure 1.**
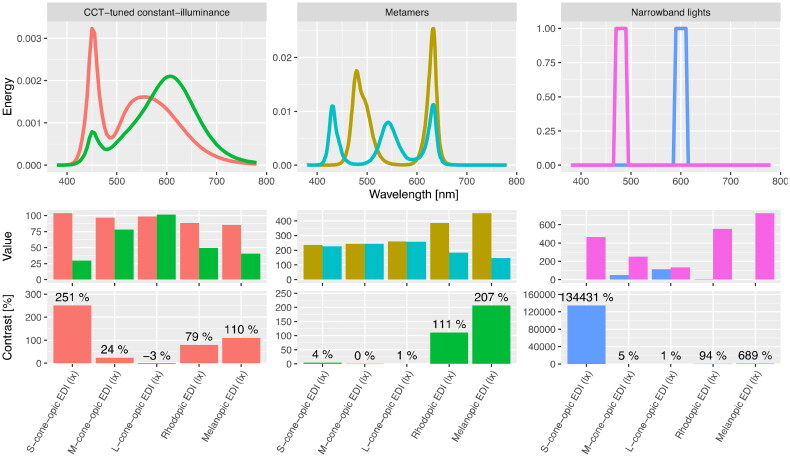
Examples of stimulus conditions and the quantification of their effects in an α-opic framework. Metameric spectra are from [88]. Code to produce the figure and underlying data can be obtained from https://github.com/tscnlab/Spitschan_AnnMed_2023.

The evaluation of control/placebo conditions in the α-opic sense, i.e. computing the predicted effects on different photoreceptor classes, goes hand in hand with selecting the conditions. Importantly, any stimulus conditions used in a study should be carefully characterized using spectroradiometric measurements. While data sheets produced by manufacturers may offer some preliminary information on spectral power distribution and other characteristics, the gold standard involves *in situ* measurements of corneal light exposure. Guides for these measurements have been developed [20,85,86], and more recently, the ENLIGHT Checklist [87] provides a systematic way for planning these measurements, to produce common reporting across different studies (Figure 1).

Unfortunately, the use of inexpensive photometers reporting lux is in many situations not sufficient, as they can only provide a limited view on the retinal, α-opic effects of a given light exposure. Research labs with limited resources should seek collaborators at their institution with access to spectroradiometers or collaborate with experts at other institutions.

Importantly, as light sources are generally subject to fluctuations with temperature or over time with ageing, it is advisable to take measurements repeatedly of the light stimuli. In order to ensure that stimulus conditions are indeed reached at the level of the participants’ eye, wearable light loggers at or near the corneal plane should be used to track personalized light exposure [89–97].

#### α-opic calculations

The calculations for converting spectral measurements into α-opic quantities are relatively simple, involving only a weighted sum (turning into α-opic radiance/irradiance) or a weighted sum scaled by a factor (turning into α-opic equivalent daylight illuminance/luminance). Once spectral measurements have been taken, they can conveniently be converted into α-opic quantities using the CIE Toolbox or luox [17] (https://luox.app/). luox provides a convenient way for making available and sharing spectra (see below under Section ‘Step 4: reporting’).

Importantly, in the evaluation of control/placebo conditions, the key is the comparison between active and control/placebo condition. This can be expressed as contrast or ratio between the α-opic activations of the active and control/placebo condition, respectively. Mathematically, the ratio between the modulation and the background is given as $\frac{E_{\text{Modulation}}}{E_{\text{Background}}}$. The contrast is given as $\frac{E_{\text{Modulation}}-E_{\text{Background}}}{E_{\text{Background}}}$ or $100\times \frac{E_{\text{Modulation}}-E_{\text{Background}}}{E_{\text{Background}}}$ (in %). Trivially, ratio and contrast simply differ by the number 1. A ratio of 1x, corresponds to 0, or 0%, contrast. A ratio of 2x corresponds to 1, or 100%, contrast. In some cases involving large differences in activation, it may be advisable to consider the different logarithmically with base 10, so as to give the orders of magnitude by which stimuli differ.

#### Visualizations

The calculated ratio and/or contrast can be conveniently visualized using bar plots (see Figure 1).

#### Physiological validation

In addition to nominal calculations to confirm the parameters chosen in Section ‘Step 1: Selection’, it is advisable to examine how the different conditions – active and control/placebo – indeed influence the retinal irradiance. A convenient method for doing so is the pupillary light reflex, which is driven by all photoreceptors, but primarily by the melanopsin-encoded ipRGC response [98]. The logic of such a physiological validation is as follows: the stimulus characteristics – e.g. in intensity and wavelength – should yield different physiological fingerprints as early as in the pupil response. If no difference in pupil response can be found, it is not clear that the targeted physiological system is indeed targeted.

### Step 4: reporting

The ENLIGHT Checklist [87] should be followed to report the light interventions in general. These represent the consensus of a group of international researchers. It is advised that the quantities (see Section ‘α-opic calculations’) and visualizations (see ‘Visualizations’) are included either in the main manuscript or in the supplement. Depending on the nature of the study, standard reporting guidelines and checklists must be followed, e.g. CONSORT [99]. The EQUATOR Network (https://www.equator-network.org/) is an excellent resource for this.

## Conclusions

Demonstrating the efficacy of light interventions requires the careful construction of control conditions. Here, we examined several strategies for constructing control conditions in studies employing light interventions, including dim-light, darkness, different-wavelength, spectral-composition controls and metameric/silent-substitution conditions. For clinical researchers interested in designing and including control conditions, we provided a practical guide for selecting, implementing, evaluating and reporting light interventions, including their controls.

## Data Availability

Code to produce the figure and underlying data can be obtained from https://github.com/tscnlab/Spitschan_AnnMed_2023.

## References

[1] Lam RW, Levitt AJ, Levitan RD, et al. Efficacy of bright light treatment, fluoxetine, and the combination in patients with nonseasonal major depressive disorder: a randomized clinical trial. JAMA Psychiatry. 2016;73(1):1–10. doi: 10.1001/jamapsychiatry.2015.2235. 26580307

[2] Hirakawa H, Terao T, Muronaga M, et al. Adjunctive bright light therapy for treating bipolar depression: a systematic review and meta-analysis of randomized controlled trials. Brain Behav. 2020;10(12):e01876. doi: 10.1002/brb3.1876. 33034127 PMC7749573

[3] Penders TM, Stanciu CN, Schoemann AM, et al. Bright light therapy as augmentation of pharmacotherapy for treatment of depression: a systematic review and meta-analysis. Prim Care Companion CNS Disord. 2016;18(5). doi: 10.4088/PCC.15r01906. 27835725

[4] Pail G, Huf W, Pjrek E, et al. Bright-light therapy in the treatment of mood disorders. Neuropsychobiology. 2011;64(3):152–162. doi: 10.1159/000328950. 21811085

[5] van Maanen A, Meijer AM, van der Heijden KB, et al. The effects of light therapy on sleep problems: a systematic review and meta-analysis. Sleep Med Rev. 2016;29:52–62. doi: 10.1016/j.smrv.2015.08.009. 26606319

[6] Beauchamp MT, Lundgren JD. A systematic review of bright light therapy for eating disorders. Prim Care Companion CNS Disord. 2016;18(5). doi: 10.4088/PCC.16r02008. 27835724

[7] Nussbaumer-Streit B, Forneris CA, Morgan LC, et al. Light therapy for preventing seasonal affective disorder. Cochrane Database Syst Rev. 2019;6(6):CD011271. 30883670 10.1002/14651858.CD011269.pub3 PMC6422319

[8] Do MTH. Melanopsin and the intrinsically photosensitive retinal ganglion cells: biophysics to behavior. Neuron. 2019;104(2):205–226. doi: 10.1016/j.neuron.2019.07.016. 31647894 PMC6944442

[9] Provencio I, Rodriguez IR, Jiang G, et al. A novel human opsin in the inner retina. J Neurosci. 2000;20(2):600–605. doi: 10.1523/JNEUROSCI.20-02-00600.2000. 10632589 PMC6772411

[10] Rollag MD, Berson DM, Provencio I. Melanopsin, ganglion-cell photoreceptors, and mammalian photoentrainment. J Biol Rhythms. 2003;18(3):227–234. doi: 10.1177/0748730403018003005. 12828280

[11] Berson DM, Dunn FA, Takao M. Phototransduction by retinal ganglion cells that set the circadian clock. Science. 2002;295(5557):1070–1073. doi: 10.1126/science.1067262. 11834835

[12] Hattar S, Liao HW, Takao M, et al. Melanopsin-containing retinal ganglion cells: architecture, projections, and intrinsic photosensitivity. Science. 2002;295(5557):1065–1070. doi: 10.1126/science.1069609. 11834834 PMC2885915

[13] Lucas RJ, Hattar S, Takao M, et al. Diminished pupillary light reflex at high irradiances in melanopsin-knockout mice. Science. 2003;299(5604):245–247. doi: 10.1126/science.1077293. 12522249

[14] Spitschan M. Melanopsin contributions to non-visual and visual function. Curr Opin Behav Sci. 2019;30:67–72. doi: 10.1016/j.cobeha.2019.06.004. 31396546 PMC6687502

[15] Rushton WAH. Review lecture, pigments and signals in colour vision. J Physiol. 1972;220(3):1P–31P. 4336741 10.1113/jphysiol.1972.sp009719 PMC1331666

[16] CIE. CIE S 026/E:2018: CIE system for metrology of optical radiation for ipRGC-Influenced responses to light. Vienna: CIE Central Bureau; 2018.

[17] Spitschan M, Mead J, Roos C, et al. Luox: validated reference open-access and open-source web platform for calculating and sharing physiologically relevant quantities for light and lighting. Wellcome Open Res. 2021;6:69. doi: 10.12688/wellcomeopenres.16595.3. 34017925 PMC8095192

[18] Price LLA, Blattner P. Circadian and visual photometry. Prog Brain Res. 2022;273(1):1–11. doi: 10.1016/bs.pbr.2022.02.014. 35940711

[19] Schlangen LJM, Price LLA. The lighting environment, its metrology, and non-visual responses. Front Neurol. 2021;12:624861. doi: 10.3389/fneur.2021.624861. 33746879 PMC7970181

[20] Spitschan M, Stefani O, Blattner P, et al. How to report light exposure in human chronobiology and sleep research experiments. Clocks Sleep. 2019;1(3):280–289. doi: 10.3390/clockssleep1030024. 31281903 PMC6609447

[21] Wirz-Justice A. Light therapy for depression: present status, problems, and perspectives. Psychopathology. 1986;19(Suppl. 2):136–141. doi: 10.1159/000285145. 3554299

[22] Rosenthal NE, Sack DA, Skwerer RG, et al. Phototherapy for seasonal affective disorder. J Biol Rhythms. 1988;3(2):101–120. doi: 10.1177/074873048800300202. 2979634

[23] Brown WA. Is light treatment a placebo? Psychopharmacol Bull. 1990;26(4):527–530. 2087547

[24] Eastman CI. What the placebo literature can tell us about light therapy for SAD. Psychopharmacol Bull. 1990;26(4):495–504. 2087542

[25] Stewart J. Placebos in evaluating light therapy for seasonal affective disorder. Psychopharmacol Bull. 1990;26(4):525–526. 2087546

[26] Terman M. Problems and prospects for use of bright light as a therapeutic intervention. In: Light and biological rhythms in man. Elsevier; 1993 p. 421–36. Available from: https://linkinghub.elsevier.com/retrieve/pii/B9780080422794500352

[27] Groß A, Möller HJ. Problem des Placeboeffektes bei Lichttherapie. In: Kasper S, Möller HJ, editors. Herbst-/Winterdepression und Lichttherapie . Vienna: Springer; 2004. p. 119–124. Available from: 10.1007/978-3-7091-0592-4_13

[28] Eastman C. Stories from a life studying circadian rhythms and sleep. Sleep Advances. 2023;4(1) 10.1093/sleepadvances/zpad040 PMC10710544 38084297

[29] Walbeek TJ, Harrison EM, Gorman MR, et al. Naturalistic intensities of light at night: a review of the potent effects of very dim light on circadian responses and considerations for translational research. Front Neurol. 2021;12:625334. doi: 10.3389/fneur.2021.625334. 33597916 PMC7882611

[30] Allen AE, Hazelhoff EM, Martial FP, et al. Exploiting metamerism to regulate the impact of a visual display on alertness and melatonin suppression independent of visual appearance. Sleep. 2018;41(8):zsy100. 29788219 10.1093/sleep/zsy100 PMC6093320

[31] Souman JL, Borra T, de Goijer I, et al. Spectral tuning of white light allows for strong reduction in melatonin suppression without changing illumination level or color temperature. J Biol Rhythms. 2018;33(4):420–431. 29984614 10.1177/0748730418784041

[32] de Zeeuw J, Papakonstantinou A, Nowozin C, et al. Living in biological darkness: objective sleepiness and the pupillary light responses are affected by different metameric lighting conditions during daytime. J Biol Rhythms. 2019;34(4):410–431. 31156018 10.1177/0748730419847845 PMC6637815

[33] Zandi B, Stefani O, Herzog A, et al. Optimising metameric spectra for integrative lighting to modulate the circadian system without affecting visual appearance. Sci Rep. 2021;11(1):23188. 34848762 10.1038/s41598-021-02136-y PMC8633386

[34] Blume C, Niedernhuber M, Spitschan M, et al. Melatonin suppression does not automatically alter sleepiness, vigilance, sensory processing, or sleep. Sleep. 2022;45(11):zsac199. doi: 10.1093/sleep/zsac199. 35998110 PMC9644120

[35] Estévez O, Spekreijse H. The “silent substitution” method in visual research. Vision Res. 1982;22(6):681–691. doi: 10.1016/0042-6989(82)90104-3. 7112962

[36] Spitschan M, Woelders T. The method of silent substitution for examining melanopsin contributions to pupil control. Front Neurol. 2018;9:941. doi: 10.3389/fneur.2018.00941. 30538662 PMC6277556

[37] Lewy AJ, Wehr TA, Goodwin FK, et al. Light suppresses melatonin secretion in humans. Science. 1980;210(4475):1267–1269. doi: 10.1126/science.7434030. 7434030

[38] Brainard GC, Hanifin JP, Greeson JM, et al. Action spectrum for melatonin regulation in humans: evidence for a novel circadian photoreceptor. J Neurosci. 2001;21(16):6405–6412. doi: 10.1523/JNEUROSCI.21-16-06405.2001. 11487664 PMC6763155

[39] Thapan K, Arendt J, Skene DJ. An action spectrum for melatonin suppression: evidence for a novel non-rod, non-cone photoreceptor system in humans. J Physiol. 2001;535(Pt 1):261–267. 11507175 10.1111/j.1469-7793.2001.t01-1-00261.x PMC2278766

[40] Brown TM. Melanopic illuminance defines the magnitude of human circadian light responses under a wide range of conditions. J Pineal Res. 2020;69(1):e12655. 32248548 10.1111/jpi.12655

[41] Khalsa SBS, Jewett ME, Cajochen C, et al. A phase response curve to single bright light pulses in human subjects. J Physiol. 2003;549(Pt 3):945–952. 12717008 10.1113/jphysiol.2003.040477 PMC2342968

[42] Minors DS, Waterhouse JM, Wirz-Justice A. A human phase-response curve to light. Neurosci Lett. 1991;133(1):36–40. 1791996 10.1016/0304-3940(91)90051-t

[43] Rüger M, St Hilaire MA, Brainard GC, et al. Human phase response curve to a single 6.5 h pulse of short-wavelength light. J Physiol. 2013;591(1):353–363. doi: 10.1113/jphysiol.2012.239046. 23090946 PMC3630790

[44] Dodson ER, Zee PC. Therapeutics for circadian rhythm sleep disorders. Sleep Med Clin. 2010;5(4):701–715. 21243069 10.1016/j.jsmc.2010.08.001 PMC3020104

[45] Sack RL, Auckley D, Auger RR, et al. Circadian rhythm sleep disorders: part II, advanced sleep phase disorder, delayed sleep phase disorder, free-running disorder, and irregular sleep-wake rhythm. An American Academy of Sleep Medicine Review. Sleep. 2007;30(11):1484–1501. 18041481 10.1093/sleep/30.11.1484 PMC2082099

[46] Duffy JF, Kronauer RE, Czeisler CA. Phase-shifting human circadian rhythms: influence of sleep timing, social contact and light exposure. J Physiol. 1996;495(Pt 1):289–297. doi: 10.1113/jphysiol.1996.sp021593. 8866371 PMC1160744

[47] Youngstedt SD, Elliott JA, Kripke DF. Human circadian phase–response curves for exercise. J Physiol. 2019;597(8):2253–2268. doi: 10.1113/JP276943. 30784068 PMC6462487

[48] Youngstedt SD, Kline CE, Elliott JA, et al. Circadian phase-shifting effects of bright light, exercise, and bright light + exercise. J Circadian Rhythms. 2016;14:2. 27103935 10.5334/jcr.137 PMC4834751

[49] Wehrens SMT, Christou S, Isherwood C, et al. Meal timing regulates the human circadian system. Curr Biol. 2017;27(12):1768–1775.e3. doi: 10.1016/j.cub.2017.04.059. 28578930 PMC5483233

[50] Terman M, Terman JS. Controlled trial of naturalistic dawn simulation and negative air ionization for seasonal affective disorder. Am J Psychiatry. 2006;163(12):2126–2133. 17151164 10.1176/ajp.2006.163.12.2126

[51] Terman M, Terman JS, Ross DC. A controlled trial of timed bright light and negative air ionization for treatment of winter depression. Arch Gen Psychiatry. 1998;55(10):875–882. 9783557 10.1001/archpsyc.55.10.875

[52] Goel N, Terman M, Su Terman J, et al. Controlled trial of bright light and negative air ions for chronic depression. Psychol Med. 2005;35(7):945–955. 16045061 10.1017/s0033291705005027

[53] Flory R, Ametepe J, Bowers B. A randomized, placebo-controlled trial of bright light and high-density negative air ions for treatment of seasonal affective disorder. Psychiatry Res. 2010;177(1–2):101–108. 20381162 10.1016/j.psychres.2008.08.011

[54] Bowers B, Flory R, Ametepe J, et al. Controlled trial evaluation of exposure duration to negative air ions for the treatment of seasonal affective disorder. Psychiatry Res. 2018;259:7–14. 29024857 10.1016/j.psychres.2017.08.040

[55] Perez V, Alexander DD, Bailey WH. Air ions and mood outcomes: a review and meta-analysis. BMC Psychiatry. 2013;13(1):29. 23320516 10.1186/1471-244X-13-29 PMC3598548

[56] Terman M, Schlager D, Fairhurst S, et al. Dawn and dusk simulation as a therapeutic intervention. Biol Psychiatry. 1989;25(7):966–970. 2720008 10.1016/0006-3223(89)90276-x

[57] Norden MJ, Avery DH. A controlled study of dawn simulation in subsyndromal winter depression. Acta Psychiatr Scand. 1993;88(1):67–71. 8372698 10.1111/j.1600-0447.1993.tb03415.x

[58] Avery DH, Bolte MAP, Wolfson JK, et al. Dawn simulation compared with a dim red signal in the treatment of winter depression. Biol Psychiatry. 1994;36(3):180–188. 7948455 10.1016/0006-3223(94)91223-8

[59] Lingjaerde O, Føreland AR, Dankertsen J. Dawn simulation vs. lightbox treatment in winter depression: a comparative study. Acta Psychiatr Scand. 1998;98(1):73–80. 9696518 10.1111/j.1600-0447.1998.tb10045.x

[60] Meesters Y. Case study: dawn simulation as maintenance treatment in a nine-year-old patient with seasonal affective disorder. J Am Acad Child Adolesc Psychiatry. 1998;37(9):986–988. doi: 10.1097/00004583-199809000-00019. 9735618

[61] Danilenko KV, Wirz-Justice A, Kräuchi K, et al. Phase advance after one or three simulated dawns in humans. Chronobiol Int. 2000;17(5):659–668. doi: 10.1081/cbi-100101072. 11023213

[62] Danilenko KV, Wirz-Justice A, Kräuchi K, et al. The human circadian pacemaker can see by the dawn’s early light. J Biol Rhythms. 2000;15(5):437–446. doi: 10.1177/074873000129001521. 11039921

[63] Avery DH, Eder DN, Bolte MA, et al. Dawn simulation and bright light in the treatment of SAD: a controlled study. Biol Psychiatry. 2001;50(3):205–216. doi: 10.1016/S0006-3223(01)01200-8. 11513820

[64] Noguchi H, Sakaguchi T, Shirakawa S, et al. Effects of simulated dawn lighting on awakening. J Illum Eng Soc. 2001;30(1):49–56. doi: 10.1080/00994480.2001.10748333.

[65] Fontana Gasio P, Kräuchi K, Cajochen C, et al. Dawn–dusk simulation light therapy of disturbed circadian rest–activity cycles in demented elderly. Exp Gerontol. 2003;38(1–2):207–216. 12543279 10.1016/s0531-5565(02)00164-x

[66] Leppämäki S, Meesters Y, Haukka J, et al. Effect of simulated dawn on quality of sleep – a community-based trial. BMC Psychiatry. 2003;3(1):14. 14577838 10.1186/1471-244X-3-14 PMC270037

[67] Thorn L, Hucklebridge F, Esgate A, et al. The effect of dawn simulation on the cortisol response to awakening in healthy participants. Psychoneuroendocrinology. 2004;29(7):925–930. 15177708 10.1016/j.psyneuen.2003.08.005

[68] Terman M, Terman JS. Circadian rhythm phase advance with dawn simulation treatment for winter depression. J Biol Rhythms. 2010;25(4):297–301. 20679499 10.1177/0748730410374000

[69] Gabel V, Maire M, Reichert CF, et al. Effects of artificial dawn and morning blue light on daytime cognitive performance, well-being. Chronobiol Int. 2013;30(8):988–997. 23841684 10.3109/07420528.2013.793196

[70] Thompson A, Jones H, Gregson W, et al. Effects of dawn simulation on markers of sleep inertia and post-waking performance in humans. Eur J Appl Physiol. 2014;114(5):1049–1056. 24509892 10.1007/s00421-014-2831-z

[71] Tonetti L, Fabbri M, Erbacci A, et al. Effects of dawn simulation on attentional performance in adolescents. Eur J Appl Physiol. 2015;115(3):579–587. 25351789 10.1007/s00421-014-3033-4

[72] Viola AU, Gabel V, Chellappa SL, et al. Dawn simulation light: a potential cardiac events protector. Sleep Med. 2015;16(4):457–461. doi: 10.1016/j.sleep.2014.12.016. 25813092

[73] Bromundt V, Wirz-Justice A, Boutellier M, et al. Effects of a dawn–dusk simulation on circadian rest–activity cycles, sleep, mood and well-being in dementia patients. Exp Gerontol. 2019;124:110641. 31252161 10.1016/j.exger.2019.110641

[74] Gabel V, Miglis M, Zeitzer JM. Effect of artificial dawn light on cardiovascular function, alertness, and balance in middle-aged and older adults. Sleep. 2020;43(10):zsaa082. doi: 10.1093/sleep/zsaa082. 32307533

[75] Klein T, Martens H, Dijk DJ, et al. Circadian sleep regulation in the absence of light perception: chronic non-24-hour circadian rhythm sleep disorder in a blind man with a regular 24-hour sleep–wake schedule. Sleep. 1993;16(4):333–343. doi: 10.1093/sleep/16.4.333. 8341894

[76] Czeisler CA, Shanahan TL, Klerman EB, et al. Suppression of melatonin secretion in some blind patients by exposure to bright light. N Engl J Med. 1995;332(1):6–11. doi: 10.1056/NEJM199501053320102. 7990870

[77] Klerman EB, Zeitzer JM, Duffy JF, et al. Absence of an increase in the duration of the circadian melatonin secretory episode in totally blind human subjects. J Clin Endocrinol Metab. 2001;86(7):3166–3170. 11443183 10.1210/jcem.86.7.7659

[78] Klerman EB, Shanahan TL, Brotman DJ, et al. Photic resetting of the human circadian pacemaker in the absence of conscious vision. J Biol Rhythms. 2002;17(6):548–555. doi: 10.1177/0748730402238237. 12465888

[79] Zaidi FH, Hull JT, Peirson SN, et al. Short-wavelength light sensitivity of circadian, pupillary, and visual awareness in humans lacking an outer retina. Curr Biol. 2007;17(24):2122–2128. 18082405 10.1016/j.cub.2007.11.034 PMC2151130

[80] Vandewalle G, Collignon O, Hull JT, et al. Blue light stimulates cognitive brain activity in visually blind individuals. J Cogn Neurosci. 2013;25(12):2072–2085. doi: 10.1162/jocn_a_00450. 23859643 PMC4497579

[81] Hull JT, Czeisler CA, Lockley SW. Suppression of melatonin secretion in totally visually blind people by ocular exposure to white light. Ophthalmology. 2018;125(8):1160–1171. 29625838 10.1016/j.ophtha.2018.01.036

[82] Vandewalle G, van Ackeren MJ, Daneault V, et al. Light modulates oscillatory alpha activity in the occipital cortex of totally visually blind individuals with intact non-image-forming photoreception. Sci Rep. 2018;8(1):16968. 30446699 10.1038/s41598-018-35400-9 PMC6240048

[83] Spitschan M, Garbazza C, Kohl S, et al. Sleep and circadian phenotype in people without cone-mediated vision: a case series of five CNGB3 and two CNGA3 patients. Brain Commun. 2021;3(3):fcab159. 34447932 10.1093/braincomms/fcab159 PMC8385249

[84] Madsen HØ, Ba-Ali S, Hageman I, et al. Light therapy for seasonal affective disorder in visual impairment and blindness – a pilot study. Acta Neuropsychiatr. 2021;33(4):191–199. 33658092 10.1017/neu.2021.6

[85] Knoop M, Broszio K, Diakite A, et al. Methods to describe and measure lighting conditions in experiments on non-image-forming aspects. LEUKOS. 2019;15(2–3):163–179. doi: 10.1080/15502724.2018.1518716.

[86] Veitch JA, Knoop M. CIE TN 011:2020 What to document and report in studies of ipRGC-influenced responses to light . International Commission on Illumination (CIE); 2020. Available from: http://cie.co.at/publications/what-document-and-report-studies-iprgc-influenced-responses-light

[87] Spitschan M, Kervezee L, Lok R, et al. ENLIGHT: a consensus checklist for reporting laboratory-based studies on the non-visual effects of light in humans. EBioMedicine. 2023;98:104889. 38043137 10.1016/j.ebiom.2023.104889 PMC10704221

[88] Schöllhorn I, Stefani O, Lucas RJ, et al. Melanopic irradiance defines the impact of evening display light on sleep latency, melatonin and alertness. Commun Biol. 2023;6(1):228. 36854795 10.1038/s42003-023-04598-4 PMC9974389

[89] Spitschan M, Smolders K, Vandendriessche B, et al. Verification, analytical validation and clinical validation (V3) of wearable dosimeters and light loggers. Digit Health. 2022;8:20552076221144858. 36601285 10.1177/20552076221144858 PMC9806438

[90] Balajadia E, Garcia S, Stampfli J, et al. Usability and acceptability of a corneal-plane α-opic light logger in a 24-hour field trial. *Digit Biomark* 2023;7(1):139–149. 37901367 10.1159/000531404 PMC10601946

[91] Stampfli J, Schrader B, Di Battista C, et al. The light-dosimeter: a new device to help advance research on the non-visual responses to light. Light Res Technol. 2023;55(4–5):474–486. 37469656 10.1177/14771535221147140 PMC10353031

[92] Okudaira N, Kripke DF, Webster JB. Naturalistic studies of human light exposure. Am J Physiol. 1983;245(4):R613–R615. 6624956 10.1152/ajpregu.1983.245.4.R613

[93] Savides TJ, Messin S, Senger C, et al. Natural light exposure of young adults. Physiol Behav. 1986;38(4):571–574. 3823171 10.1016/0031-9384(86)90427-0

[94] Aarts MPJ, van Duijnhoven J, Aries MBC, et al. Performance of personally worn dosimeters to study non-image forming effects of light: assessment methods. Build Environ. 2017;117:60–72. doi: 10.1016/j.buildenv.2017.03.002.

[95] Hartmeyer S, Webler F, Andersen M. Towards a framework for light-dosimetry studies: methodological considerations. Light Res Technol. 2022;55:377–399.

[96] Hartmeyer S, Andersen M. Towards a framework for light-dosimetry studies: quantification metrics. Light Res Technol. 2023. doi: 10.1177/14771535231170500.

[97] Hubalek S, Brink M, Schierz C. Office workers’ daily exposure to light and its influence on sleep quality and mood. Light Res Technol. 2010;42(1):33–50. doi: 10.1177/1477153509355632.

[98] Spitschan M. Photoreceptor inputs to pupil control. Journal of Vision. 2019;19(9):5 10.1167/19.9.5 PMC6699792 31415056

[99] Schulz KF, Altman DG, Moher D, et al. CONSORT 2010 statement: updated guidelines for reporting parallel group randomised trials. BMJ. 2010;340(1):c332. 20332509 10.1136/bmj.c332 PMC2844940

